# Bayesian SIR model with change points with application to the Omicron wave in Singapore

**DOI:** 10.1038/s41598-022-25473-y

**Published:** 2022-12-02

**Authors:** Jiaqi Gu, Guosheng Yin

**Affiliations:** Department of Statistics and Actuarial Science, The University of Hong Kong, Hong Kong, China

**Keywords:** Infectious diseases, Statistics

## Abstract

The Omicron variant has led to a new wave of the COVID-19 pandemic worldwide, with unprecedented numbers of daily confirmed new cases in many countries and areas. To analyze the impact of society or policy changes on the development of the Omicron wave, the stochastic susceptible-infected-removed (SIR) model with change points is proposed to accommodate the situations where the transmission rate and the removal rate may vary significantly at change points. Bayesian inference based on a Markov chain Monte Carlo algorithm is developed to estimate both the locations of change points as well as the transmission rate and removal rate within each stage. Experiments on simulated data reveal the effectiveness of the proposed method, and several stages are detected in analyzing the Omicron wave data in Singapore.

## Introduction

The ongoing worldwide COVID-19 pandemic caused by the SARS-CoV-2 virus has spread to over 200 countries and areas with more than 523 million confirmed cases and around 7 million deaths by May 2022. Although more than 11 billion doses of vaccine have been administered, several variants of SARS-CoV-2 have appeared with faster transmission and greater virulence and led to diminished effectiveness of developed vaccines, which resulted in multiple COVID-19 pandemic waves. Among all the five variants of concern listed by the World Health Organization, the Omicron variant has a higher level of transmissibility and immune escape capability^1–6^, leading to unprecedented numbers of daily confirmed new cases in many countries and areas. Due to the unique characteristics of Omicron (high transmissibility but low mortality or severe cases), existing analysis methods on the spreading of other variants are not applicable to coping with the Omicron wave of COVID-19 pandemic.

In epidemiology, the susceptible-infected-removed (SIR) model^7^ is the most popular approach to analyzing the transmission of infectious diseases. Since the beginning of the Omicron wave, a large amount of research on the SIR model or its extensions has been conducted for making inference and prediction. For example, Van Wees et al.^8^ applied the standard SIR model to predict the infection rate and hospitalization rate in South African province Gauteng, the United Kingdom and the Netherlands. To incorporate local transmission into the SIR model, Götz^9^ developed the global–local SIR model with a locality-adjusted basic reproduction number. The multi-wave SIR model proposed by Ghosh and Ghosh^10^ allows researchers to investigate the nonperiodicity of COVID-19 pandemic waves, while Khan and Atangana^11^ separated the Omicron variant from other variants in order to estimate its basic reproduction number accurately. However, all the aforementioned approaches assume that throughout a COVID-19 pandemic wave both the transmission rate and removal rate remain unchanged. This assumption is unrealistic because both individual actions for self-protection and government policies in response to the outbreak would decrease the transmission rate. In addition, more medical resources would be allocated to combat the uprise of new cases, leading to an increase of the removal rate during a pandemic wave.

To take the effect of societal changes into consideration, the stochastic SIR model with change points is proposed to analyze the evolvement of the Omicron wave of COVID-19 pandemic. Assuming that both the transmission rate and removal rate are time-varying, binomial models are proposed for the daily reported numbers of confirmed cases and removal cases. A latent indicator vector is introduced to partition a pandemic wave into several stages. Compared to existing works^12–16^ that incorporate change points to compartmental models, our model assumes that both the transmission rate and the removal rate are time-varying and can change multiple times during the study period. Instead of being constant between any pair of adjacent change points, time-varying parameters in our model are homogenous in distribution within each stage and thus are more flexible for model fitting. We develop a Markov chain Monte Carlo (MCMC) algorithm to draw posterior samples of parameters and make Bayesian inference of pandemic wave development. Experiments on simulated datasets suggest the effectiveness of the proposed method in detecting change points of a pandemic wave and the Omicron data in Singapore are analyzed to corroborate the major changes during the Omicron wave.

The rest of this paper is organized as follows. Section “The SIR model” reviews the standard SIR model. In section “Methodology”, we propose the stochastic SIR model with change points and develop an MCMC algorithm for making Bayesian inference. Experiments on simulated datasets are conducted in section “Simulations” to illustrate the performance of the proposed method. In section “Analysis of the Omicron wave in Singapore”, we analyze the Omicron wave of COVID-19 pandemic in Singapore and find several stages with different transmission rates and removal rates which match the major societal changes in Singapore. We conclude this paper in section “Conclusion”.

## The SIR model

The susceptible-infected-removed (SIR) model^7^ is the most widely used mathematical tool to model the spreading of infectious diseases. Given a closed population of *N* individuals and three possible states, susceptible (*S*), infectious (*I*) and removed (*R*, either recovered or dead), each individual is assumed to be in one state at any time. As time goes by, the state of each individual would evolve from *S* to *I* and then from *I* to *R*, implying the process that an individual gets infected and then recovers or dies. Let *S*(*t*), *I*(*t*) and *R*(*t*) denote the number of susceptible, infectious and removed individuals in the population at time point *t* ($t\ge 0$) respectively. Define $P(t)=I(t)/N$ as the proportion of infectious individuals at time *t*. Assuming that in-person contacts among individuals follow the Erdős–Rényi model^17,18^, the standard SIR model describes the flow of individuals from *S* to *I* and then from *I* to *R* by a set of ordinary differential equations without the access to individual records of infection and removal,

1 $$\begin{aligned} {\left\{ \begin{array}{ll} \displaystyle \frac{dS(t)}{dt}=-\beta S(t)P(t),\\ \displaystyle \frac{dI(t)}{dt}=\beta S(t)P(t)-\gamma I(t),\\ \displaystyle \frac{dR(t)}{dt}=\gamma I(t),\\ \end{array}\right. } \end{aligned}$$

where $\beta$ and $\gamma$ are the transmission rate parameter and the removal rate parameter respectively. It is clear that

$$\begin{aligned} \frac{dS(t)}{dt}+\frac{dI(t)}{dt}+\frac{dR(t)}{dt}=0, \end{aligned}$$

and thus the population size is fixed as $S(t)+I(t)+R(t)=N$ for $t\ge 0$.

Although the SIR model has been widely used due to its simplicity and interpretability, it cannot fit real Omicron data well. The main reason is that the SIR model assumes that both the transmission rate $\beta$ and the removal rate $\gamma$ remain unchanged throughout the whole study period. This assumption is unrealistic for the ongoing COVID-19 pandemic, where the transmission rate would decrease as individual strategies of self-protection or government policies of social distancing are implemented. In addition, the removal rate would also increase as more medical resources are allocated to cope with the pandemic and help to cure patients.

## Methodology

### The stochastic SIR model with change points

**Figure 1:**
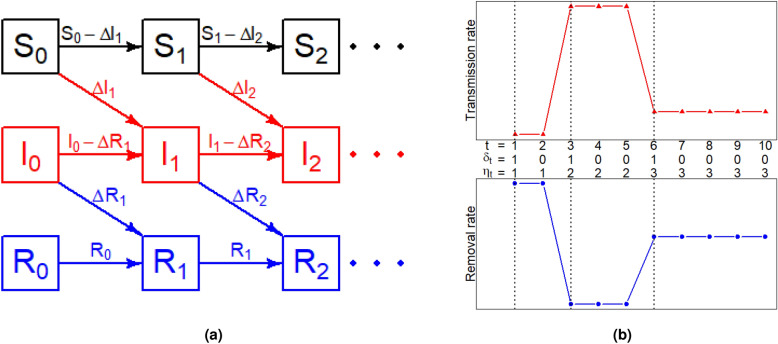
Graphical illustration of (**a**) the update of $(S_t,I_t,R_t)$; (**b**) change-points in the expected transmission rates and expected removal rates of different stages.

To model the spreading of infectious diseases with time-varying parameters, we propose the stochastic SIR model with change points. Because the numbers of confirmed cases and removed cases are reported only on a daily basis during the COVID-19 pandemic, we assume that the study period is $\{1,\ldots ,T\}$, where *T* is the length of the study period. Let ${\Delta }{{\textbf {I}}}=(\Delta I_1,\ldots ,\Delta I_T)^{\textsf{T}}$ and ${\Delta }{{\textbf {R}}}=(\Delta R_1,\ldots ,\Delta R_T)^{\textsf{T}}$ be sequences of daily reported numbers of newly infected (confirmed) cases and removed cases respectively. Given the initial state of the population $(S_0,I_0,R_0)$, we modify the SIR model in (1) and develop the discrete-time stochastic SIR model with change points as follows. For $t=1,\ldots ,T$, we assume

2 $$\begin{aligned} {\left\{ \begin{array}{ll} {\Delta I}_t\sim \text {Binomial}(S_{t-1},1-\exp (-\beta _tP_{t-1})),\\ {\Delta R}_t\sim \text {Binomial}(I_{t-1},\gamma _t),\\ S_t=S_{t-1}-{\Delta I}_t,\\ I_t=I_{t-1}+{\Delta I}_t-{\Delta R}_t,\\ R_t=R_{t-1}+{\Delta R}_t, \end{array}\right. } \end{aligned}$$

where $P_t=I_t/N$ is the proportion of infectious individuals at day *t* and $(S_t,I_t,R_t)$ are updated as shown in Fig. 1a. Time-varying parameters $\beta _t\in (0,\infty )$ and $\gamma _t\in (0,1)$ represent the transmission rate and the removal rate at day *t* respectively.

The second equation of the proposed model (2) corresponds the term $\gamma I(t)$ in (1), which indicates the number of infectious individuals being removed. Assuming that the removal of different infectious individuals are mutually independent, the number of infectious individuals being removed at day *t* ($\Delta R_t$) follows $\text {Binomial}(I_{t-1},\gamma _t)$. However, unlike the term $\beta S(t) P(t)$ in model (1), we make the following assumptions for each susceptible individual at day $t-1$: (i) The number of in-person contacts with other individuals at day *t*, $N_{\text {contact},t}$, follows $\text {Poisson}(\lambda _t)$; (ii) Given $N_{\text {contact},t}$, the number of in-person contacts with infectious individuals at day *t* follows $\text {Binomial}(N_{\text {contact},t},{P}_{t-1})$; and (iii) The probability of transmission for each in-person contact with infectious individuals is $q_t$. As a result, at day *t*, the probability for a susceptible individual getting infected is $1-\exp (-\lambda _t q_t {P}_{t-1})$. For identifiability, we reparameterize $\beta _t=\lambda _tq_t$ as the disease transmission rate at day *t*.

Unlike the original SIR model (1) where parameters $\beta$ and $\gamma$ are constant throughout the study period, our model (2) assumes that the transmission rate $\beta _t$ and the removal rate $\gamma _t$ may change significantly at several change points in the study period. The change points exist because there could be some societal changes, such as the implementation of new social distancing policies, restrictions on individual activities, and the increase of medical resources on patients, which would cause sudden changes in either the transmission rate or the removal rate or both. To depict such dynamic patterns of pandemic evolvement, we introduce a latent binary vector $\pmb {\delta }=(\delta _1,\ldots ,\delta _T)^{\textsf{T}}$, where $\delta _t=1$ indicates that day *t* is a change point and $\delta _t=0$ otherwise, and $\delta _1$ is fixed as 1 for convenience. The index of the stage that contains day *t* is $\eta _t=\sum _{u=1}^t\delta _u$ and the stage index vector is $\pmb {\eta }=(\eta _1,\ldots ,\eta _T)^{\textsf{T}}$, as illustrated in Fig. 1b. Thus, the study period is partitioned into $K=\sum _{t=1}^T\delta _t$ stages. Within each stage, parameters $(\beta _t,\gamma _t)$ are assumed to be homogeneous with the hierarchical priors,

3 $$\begin{aligned} \beta _t|\pmb {\delta },{{\text {b}}}\sim & {} \text {Exp}(b_{\small \eta _t}),\nonumber \\ \gamma _t|\pmb {\delta },{{\text {r}}}\sim & {} \text {Beta}(r_{\small \eta _t},1),\nonumber \\ b_1,\ldots ,b_K {\mathop {\sim }\limits ^{\text {i.i.d.}}}{} &\text {Gamma}(0.1,0.1),\\ r_1,\ldots ,r_K {\mathop {\sim }\limits ^{\text {i.i.d.}}}{} &\text {Gamma}(0.1,0.1),\nonumber \\ \delta _1,\ldots ,\delta _T {\mathop {\sim }\limits ^{\text {i.i.d.}}}{}& \text {Bernoulli}(p),\nonumber \end{aligned}$$

which are denoted as $\pi (\beta _t|\pmb {\delta },{{\textbf {b}}})$, $\pi (\gamma _t|\pmb {\delta },{{\textbf {r}}})$, $\pi (b_k)$, $\pi (r_k)$ and $\pi (\pmb {\delta })$ respectively, with ${{\textbf {b}}}=(b_1,\ldots ,b_K)^{\textsf{T}}$ and ${{\textbf {r}}}=(r_1,\ldots ,r_K)^{\textsf{T}}$. The expected transmission rate and the expected removal rate in stage *k* ($k=1,\ldots ,K$) are $1/b_k$ and $r_k/(1+r_k)$, respectively. The hyperparameter *p* expresses one’s prior belief on how often a change point occurs in a pandemic wave, because *p* is the probability of each time point is a change point. We fix $p=0.01$ in our experiments and also explore the sensitivity analysis with respect to *p*.

### Bayesian analysis with MCMC

To make Bayesian inference on parameters $\pmb {\delta }$, ${{\textbf {b}}}$, ${{\textbf {r}}}$, $\pmb {\beta }=(\beta _1,\ldots ,\beta _T)^{\textsf{T}}$ and $\pmb {\gamma }=(\gamma _1,\ldots ,\gamma _T)^{\textsf{T}}$, we develop an MCMC algorithm to draw samples from the posterior distribution,

4 $$\begin{aligned} \pi (\pmb {\delta },{{\text{b}}},{{\text{r}}},\pmb {\beta },\pmb {\gamma }|S_0,I_0,R_0,{\Delta }{{\textbf {I}}},{\Delta }{{\textbf {R}}}) &\propto \pi (\pmb {\delta })\prod _{k=1}^{K}\pi (b_k)\prod _{t=1}^{T}\big \{\pi (\beta _t|\pmb {\delta },{{\textbf {b}}}){\mathbb {P}}(\Delta I_t|S_{t-1},I_{t-1},\beta _t)\big \}\\&\quad \times \prod _{k=1}^{K}\pi (r_k)\prod _{t=1}^{T}\big \{\pi (\gamma _t|\pmb {\delta },{{\textbf {r}}}){\mathbb {P}}(\Delta R_t|I_{t-1},\gamma _t)\big \}, \end{aligned} $$

where the $\pi (\cdot )$ functions on the right-hand side correspond to the priors in (3), and ${\mathbb {P}}(\Delta I_t|S_{t-1},I_{t-1},\beta _t)$ and ${\mathbb {P}}(\Delta R_t|I_{t-1},\gamma _t)$ represent the binomial likelihood functions in (2). At each iteration, parameters are sequentially updated with the Gibbs sampling procedure detailed as follows.

**Figure 2:**
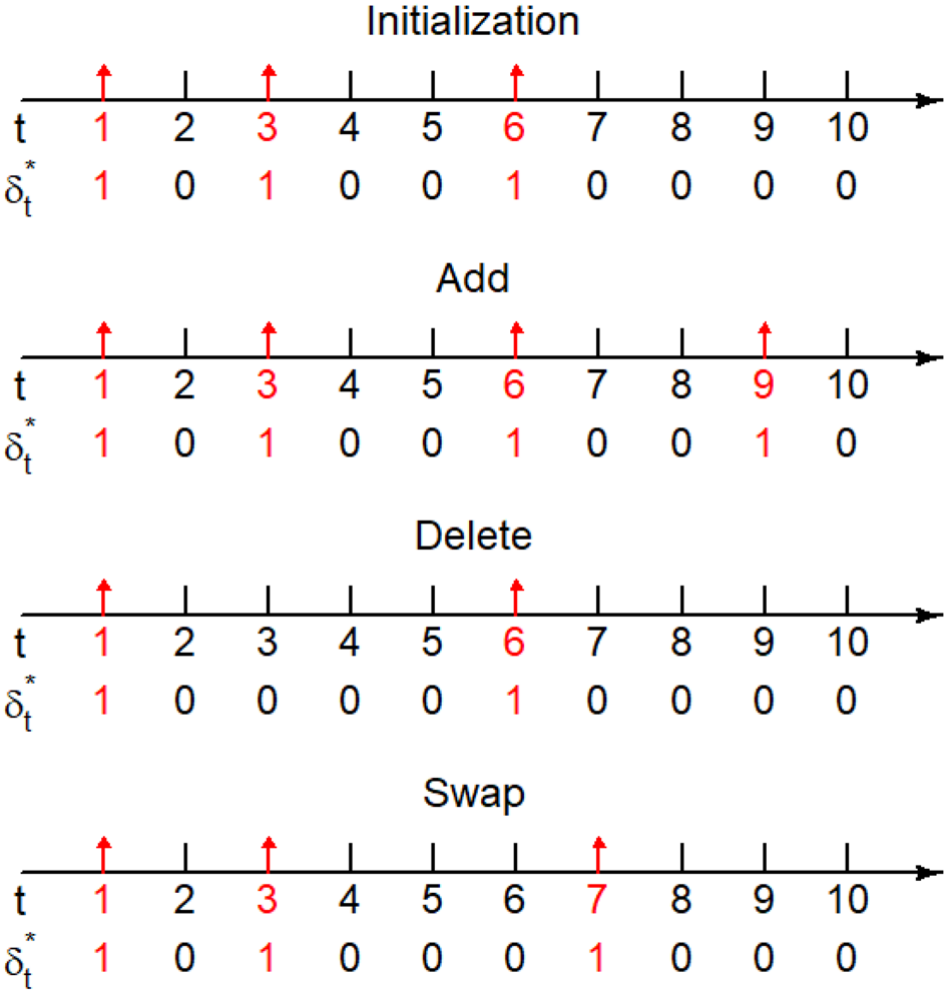
Illustration of the *add*–*delete*–*swap* algorithm. Based on the initialization $\pmb {\delta }^*=\pmb {\delta }^{(g)}=(1,0,1,0,0,1,0,0,0,0)$, the candidate $\pmb {\delta }^*$ in the $(g+1)$-th update can be obtained as: (i) (1, 0, 1, 0, 0, 1, 0, 0, 1, 0) by selecting the *add* operation and updating $\delta _9^*$ as 1; (ii) (1, 0, 0, 0, 0, 1, 0, 0, 0, 0) by selecting the *delete* operation and updating $\delta _3^*$ as 0; (iii) (1, 0, 1, 0, 0, 0, 1, 0, 0, 0) by selecting the *swap* operation and exchanging values of $\delta _6^*$ and $\delta _7^*$.

- **Update** $\pmb {\delta }$: Given current values $\pmb {\beta }^{(g)}$ and $\pmb {\gamma }^{(g)}$, $\pmb {\delta }$ is updated via an *add*–*delete*–*swap* algorithm. Initializing $\pmb {\delta }^*=\pmb {\delta }^{(g)}$, an operation is selected from {*add*, *delete*, *swap*} with probabilities,
  5 $$\begin{aligned} (p_{add },p_{delete }, p_{swap })={\left\{ \begin{array}{ll} (1,0,0),&{}\quad \text {if }K^{(g)}=1,\\ (0,1,0),&{}\quad \text {if }K^{(g)}=T,\\ (1/3,1/3,1/3),&{}\quad \text {otherwise},\\ \end{array}\right. } \end{aligned}$$
  where $K^{(g)}=\sum _{t=1}^T\delta _t^{(g)}$. If the *add* (or *delete*) operation is selected, we randomly select a $\delta ^*_t$ which is 0 (or 1) and update its value as 1 (or 0). If the *swap* operation is selected, we randomly select a pair of $(\delta ^*_t,\delta ^*_{t-1})$ with different values and exchange their values. Examples of the candidate $\pmb {\delta }^*$ obtained by different operations are shown in Fig. 2. Given the candidate $\pmb {\delta }^*$, we compute the Metropolis–Hasting ratio as
  $$\begin{aligned} m_{\text {MH}}=\frac{\pi (\pmb {\beta }^{(g)},\pmb {\gamma }^{(g)}|\pmb {\delta }^*)}{\pi (\pmb {\beta }^{(g)},\pmb {\gamma }^{(g)}|\pmb {\delta }^{(g)})}\cdot \frac{\pi (\pmb {\delta }^*)}{\pi (\pmb {\delta }^{(g)})}\cdot \frac{J(\pmb {\delta }^{(g)}|\pmb {\delta }^*)}{J(\pmb {\delta }^*|\pmb {\delta }^{(g)})}, \end{aligned}$$
  where following (3) we can derive
  $$\begin{aligned} \frac{\pi (\pmb {\delta }^*)}{\pi (\pmb {\delta }^{(g)})}=&\frac{p^{\sum _{t=1}^T\delta _t^*}(1-p)^{T-\sum _{t=1}^T\delta _t^*}}{p^{\sum _{t=1}^T\delta _t^{(g)}}(1-p)^{T-\sum _{t=1}^T\delta _t^{(g)}}}=\left( \frac{p}{1-p}\right) ^{\sum _{t=1}^T(\delta _t^*-\delta _t^{(g)})}, \end{aligned}$$
  $$\begin{aligned}&\pi (\pmb {\beta }^{(g)},\pmb {\gamma }^{(g)}|\pmb {\delta })\\&\quad =\int _{b_1=0}^\infty \ldots \int _{b_K=0}^\infty \int _{r_1=0}^\infty \ldots \int _{r_K=0}^\infty \left\{ \prod _{k=1}^{K}\pi (b_k)\prod _{t=1}^{T}\pi (\beta _t^{(g)}|\pmb {\delta },{{\text {b}}})\prod _{k=1}^{K}\pi (r_k)\prod _{t=1}^{T}\pi (\gamma _t^{(g)}|\pmb {\delta },{{\text {r}}})\right\} dr_K\ldots dr_1db_K\ldots db_1\\&\quad =\prod _{k=1}^{K}\left\{ \int _{0}^\infty \pi (b_k)\prod _{t:\eta _t=k}\pi (\beta _t^{(g)}|\pmb {\delta },{{\text {b}}})db_k\right\} \prod _{k=1}^{K}\left\{ \int _{0}^\infty \pi (r_k)\prod _{t:\eta _t=k}\pi (\gamma _t^{(g)}|\pmb {\delta },{{\text {r}}})dr_k\right\} \\&\quad =\prod _{k=1}^{K}\left\{ \int _{0}^\infty \frac{0.1^{0.1}b_k^{-0.9}e^{-0.1b_k}}{\Gamma (0.1)}\times \prod _{t:\eta _t=k}b_ke^{-\beta _t^{(g)}b_k}db_k\right\} \prod _{k=1}^{K}\left\{ \int _{0}^\infty \frac{0.1^{0.1}r_k^{-0.9}e^{-0.1r_k}}{\Gamma (0.1)}\times \prod _{t:\eta _t=k}r_k(\gamma _{t}^{(g)})^{r_k-1}dr_k\right\} \\ \propto&\prod _{k=1}^{K}\left\{ \frac{\Gamma (0.1+\sum _{t=1}^T {\mathbb {I}}(\eta _t=k))}{\{0.1+\sum _{t=1}^T \beta ^{(g)}_t{\mathbb {I}}(\eta _t=k)\}^{0.1+\sum _{t=1}^T {\mathbb {I}}(\eta _t=k)}}\right\} \prod _{k=1}^{K}\left\{ \frac{\Gamma (0.1+\sum _{t=1}^T {\mathbb {I}}(\eta _t=k))}{\{0.1+\sum _{t=1}^T -\log \gamma ^{(g)}_t{\mathbb {I}}(\eta _t=k)\}^{0.1+\sum _{t=1}^T {\mathbb {I}}(\eta _t=k)}}\right\} , \end{aligned}$$
  with $\Gamma (\cdot )$ being the gamma function, and the proposal of the Metropolis–Hasting algorithm is
  $$\begin{aligned} \frac{J(\pmb {\delta }^{(g)}|\pmb {\delta }^*)}{J(\pmb {\delta }^*|\pmb {\delta }^{(g)})}={\left\{ \begin{array}{ll} 1,&{}\quad \text {if }\sum _{t=1}^T\delta _t^*=\sum _{t=1}^T\delta _t^{(g)},\\ 3/(T-1),&{}\quad \text {if }(\sum _{t=1}^T\delta _t^*,\sum _{t=1}^T\delta _t^{(g)})=(1,2)\text { or }(T,T-1),\\ (T-1)/3,&{}\quad \text {if }(\sum _{t=1}^T\delta _t^*,\sum _{t=1}^T\delta _t^{(g)})=(2,1)\text { or }(T-1,T),\\ (\sum _{t=1}^T\delta _t^{(g)}-1)/(T-\sum _{t=1}^T\delta _t^*),&{}\quad \text {if }(\sum _{t=1}^T\delta _t^*,\sum _{t=1}^T\delta _t^{(g)})\in \{(2,3),(3,4),\ldots ,(T-2,T-1)\},\\ (T-\sum _{t=1}^T\delta _t^{(g)})/(\sum _{t=1}^T\delta _t^{*}-1),&{}\quad \text {if }(\sum _{t=1}^T\delta _t^*,\sum _{t=1}^T\delta _t^{(g)})\in \{(3,2),(4,3),\ldots ,(T-1,T-2)\}.\\ \end{array}\right. } \end{aligned}$$
  We then obtain the updated value $\pmb {\delta }^{(g+1)}$ as
  $$\begin{aligned} \pmb {\delta }^{(g+1)}={\left\{ \begin{array}{ll} \pmb {\delta }^*,&{}\quad \text {with probability }\min (1,m_{\text {MH}}),\\ \pmb {\delta }^{(g)},&{}\quad \text {with probability }1-\min (1,m_{\text {MH}}).\\ \end{array}\right. } \end{aligned}$$
  Correspondingly, we also update $K^{(g+1)}=\sum _{t=1}^T\delta _t^{(g+1)}$ and $\eta _t^{(g+1)}=\sum _{u=1}^t\delta _u^{(g+1)}$ ($t=1,\ldots ,T$).
- **Update** ${{\textbf {b}}}$ **and** ${{\textbf {r}}}$: Given current values $\pmb {\delta }^{(g+1)}$, $\pmb {\beta }^{(g)}$ and $\pmb {\gamma }^{(g)}$, we sample ${b}_k^{(g+1)}$ and ${r}_k^{(g+1)}$ from
  $$\begin{aligned}  b_k&\sim \text {Gamma}\left( 0.1+\sum _{t=1}^T {\mathbb {I}}(\eta _t^{(g+1)}=k),0.1+\sum _{t=1}^T \beta ^{(g)}_t{\mathbb {I}}(\eta _t^{(g+1)}=k)\right) ,\\r_k&\sim \text {Gamma}\left( 0.1+\sum _{t=1}^T {\mathbb {I}}(\eta _t^{(g+1)}=k),0.1+\sum _{t=1}^T -\log \gamma ^{(g)}_t{\mathbb {I}}(\eta _t^{(g+1)}=k)\right) ,\\ \end{aligned} $$
  for $k=1,\ldots ,K^{(g+1)}$, where ${\mathbb {I}}(\cdot )$ is the indicator function.
- **Update** $\pmb {\beta }$ **and** $\pmb {\gamma }$: Given current values $\pmb {\delta }^{(g+1)}$, ${{\textbf {b}}}^{(g+1)}$ and ${{\textbf {r}}}^{(g+1)}$, we sample ${\beta }_t^{(g+1)}$ and ${\gamma }_t^{(g+1)}$ from posterior densities,
  $$\begin{aligned} &\pi (\beta _t|S_{t-1},\Delta I_{t},b^{(g+1)}_{\small \eta _t^{(g+1)}})\propto \{1-\exp (-\beta _tP_{t-1})\}^{\Delta I_{t}}\exp \{-\beta _tP_{t-1}(S_{t-1}-\Delta I_{t})\}\pi (\beta _t|b^{(g+1)}_{\small \eta _t^{(g+1)}}),\\&\pi (\gamma _t|I_{t-1},\Delta R_{t},r^{(g+1)}_{\small \eta _t^{(g+1)}})\propto \gamma _t^{\Delta R_{t}}(1-\gamma _t)^{I_{t-1}-\Delta R_{t}}\pi (\gamma _t|r^{(g+1)}_{\small \eta _t^{(g+1)}}),\\ \end{aligned} $$
  for $$t=1,\ldots ,T$$.

Using the MCMC algorithm, a set of posterior samples $\{(\pmb {\delta }^{(g)},{{\textbf {b}}}^{(g)},{{\textbf {r}}}^{(g)},\pmb {\beta }^{(g)},\pmb {\gamma }^{(g)}): g=1,\ldots ,G\}$ is obtained for inference. Because the main interest lies in detection of change points, we aggregate $\pmb {\delta }^{(1)},\ldots ,\pmb {\delta }^{(G)}$ to obtain a point estimate $\hat{\pmb {\delta }}$ (or the corresponding $\hat{\pmb {\eta }}$). As the indicator vector $\pmb {\delta }$ is a binary vector with $2^{T-1}$ possible values in total, its posterior mean does not imply a partition of the study period and thus is difficult to interpret. Taking the sequential structure of $\pmb {\delta }$ into consideration, we interpret each $\pmb {\delta }^{(g)}$ (or the corresponding ${\pmb {\eta }}^{(g)}$) as a cluster of time points $1,\ldots ,T$ and obtain $\hat{\pmb {\delta }}$ by solving a clustering aggregation problem as follows.

- For each pair of time points *t* and $t'$ ($1\le t< t'\le T$), we estimate the posterior probability that no change points exist in the time period $\{t+1,\ldots , t'\}$ as
  $$\begin{aligned} {\hat{q}}_{tt'}=\frac{1}{G}\sum _{g=1}^G {\mathbb {I}}(\eta _t^{(g)}=\eta _{t'}^{(g)}), \end{aligned}$$
  where ${\mathbb {I}}(\cdot )$ is the indicator function.
- The Bayes estimator $\hat{\pmb {\delta }}$ is then obtained as
  $$\begin{aligned} \hat{\pmb {\delta }}=\arg \min _{\pmb {\delta }}\sum _{1\le t< t'\le T}\Big |{\mathbb {I}}(\eta _t=\eta _{t'})-{\hat{q}}_{tt'}\Big |, \end{aligned}$$
  $\text {where }{\eta }_t=\sum _{u=1}^t{\delta }_u$. The Bayes estimator $\hat{\pmb {\eta }}$ can also be obtained correspondingly.

Based on $\hat{\pmb {\delta }}$, we can also compute probability regions of change points. With ${\hat{K}}=\sum _{t=1}^T{\hat{\delta }}_t$, let ${\hat{\tau }}_k=\min \{t:\sum _{u=1}^t{\hat{\delta }}_t\ge k\}$ denote the *k*-th estimated change point ($k=1,\ldots ,{\hat{K}}$). The $(1-\alpha )$ highest posterior density (HPD) interval of the *k*-th change point is computed as $[{\hat{\tau }}_{lk},{\hat{\tau }}_{uk}]$, where

$$\begin{aligned} {\hat{\tau }}_{lk},{\hat{\tau }}_{uk}&=\mathop {\arg \min }_{\tau _l\le {\hat{\tau }}_k\le \tau _u}(\tau _u-\tau _l),\quad \text {s.t.}\quad \frac{1}{G}\sum _{u=\tau _l}^{\tau _u} \sum _{g=1}^G \delta _u^{(g)} \ge 1-\alpha . \end{aligned} $$

As the number of stages varies among posterior samples, we use $G^{-1}\sum _{g=1}^G {1}/{b^{(g)}_{\small \eta ^{(g)}_t}}$ and $G^{-1}\sum _{g=1}^G {r^{(g)}_{\small \eta ^{(g)}_t}}/{(1+r^{(g)}_{\small \eta ^{(g)}_t})}$ as smoothed estimators of the expected transmission rate and expected removal rate at day *t* respectively.

## Simulations

To evaluate the performance of the proposed method in detecting change points of the transmission rate and removal rate during a pandemic wave, we conduct simulated experiments by applying the developed MCMC algorithm for making Bayesian inference.

**Figure 3:**
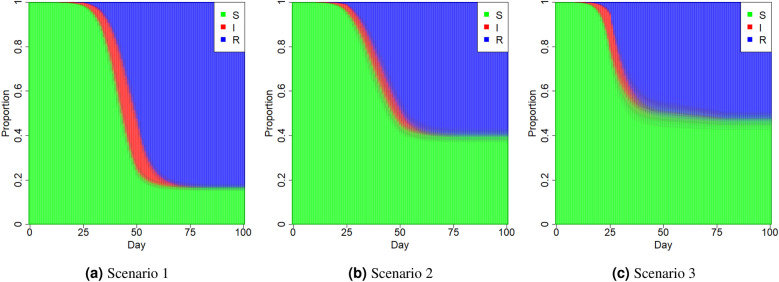
Average proportions of susceptible (S), infectious (I) and removed (R) individuals in the population at time *t* ($t=1,\ldots ,100$) under different scenarios.

### Data generating mechanism

Considering a study period of length $T=100$ and a fixed population size $N=1000000$, we partition the study period $\{1,\ldots ,T\}$ into four stages evenly. In other words, there are three true change points at $t=26$, $t=51$ and $t=76$. To mimic the dynamics of the transmission rate and removal rate during a pandemic wave, we design three scenarios with the time-varying transmission rate and removal rate as follows,

- Scenario 1:
  $$\begin{aligned} (\beta _{t},\gamma _{t})={\left\{ \begin{array}{ll} (0.3,0.05),&{}\quad t=1,\ldots ,25;\\ (0.4,0.15),&{}\quad t=26,\ldots ,50;\\ (0.25,0.2),&{}\quad t=51,\ldots ,75;\\ (0.2,0.25),&{}\quad t=76,\ldots ,100.\\ \end{array}\right. } \end{aligned}$$
- Scenario 2:
  $$\begin{aligned} (\beta _{t},\gamma _{t})={\left\{ \begin{array}{ll} (0.4,0.1),&{}\quad t=1,\ldots ,25;\\ (0.4,0.25),&{}\quad t=26,\ldots ,50;\\ (0.25,0.25),&{}\quad t=51,\ldots ,75;\\ (0.25,0.4),&{}\quad t=76,\ldots ,100.\\ \end{array}\right. } \end{aligned}$$
- Scenario 3:
  $$\begin{aligned} (\beta _{t},\gamma _{t})={\left\{ \begin{array}{ll} (0.5,0.1),&{}\quad t=1,\ldots ,25;\\ (0.3,0.3),&{}\quad t=26,\ldots ,50;\\ (0.4,0.2),&{}\quad t=51,\ldots ,75;\\ (0.2,0.4),&{}\quad t=76,\ldots ,100.\\ \end{array}\right. } \end{aligned}$$

With initial numbers $(S_0,I_0,R_0)=(N-50,50,0)$, we generate 100 datasets for each of three scenarios. Within each dataset, daily confirmed cases ${\Delta }{{\textbf {I}}}$ and daily removed cases ${\Delta }{{\textbf {R}}}$ are generated sequentially as Fig. 1a under the model (2) from $t=1$ to $t=T$. Given ${\Delta }{{\textbf {I}}}$ and ${\Delta }{{\textbf {R}}}$, we exhibit $(S_t,I_t,R_t)$ ($1\le t\le T$) in Fig. 3. It is clear that the severity of the pandemic wave is the strongest under Scenario 1 and the total number of infections is the smallest under Scenario 3.

### Bayesian analysis

We apply the MCMC algorithm proposed in section “Methodology” with hyperparameter $p=0.01$ to analyze simulated datasets. For each dataset, we discard the first 5000 iterations as burn-in and draw one posterior sample for every 10 iterations in the next 10000 iterations, leading to $G=1000$ posterior samples $(\pmb {\delta }^{(g)},{{\textbf {b}}}^{(g)},{{\textbf {r}}}^{(g)},\pmb {\beta }^{(g)},\pmb {\gamma }^{(g)})$ in total.

**Figure 4:**
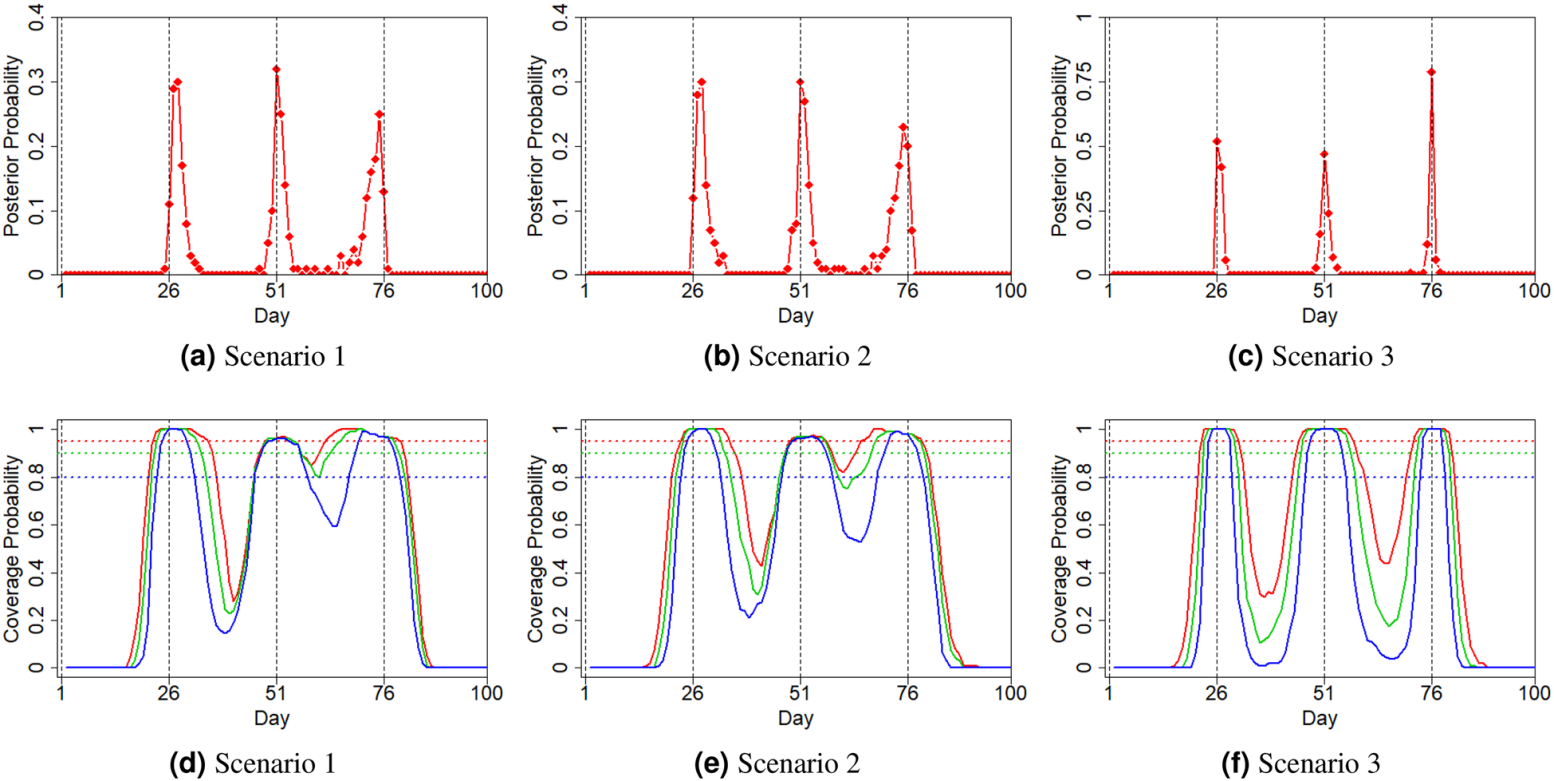
(**a**–**c**) Proportions that a change point is estimated at time *t* ($1\le t\le T$) under different scenarios; (**d**–**f**) Coverage probability of the $(1-\alpha )$ HPD intervals of change points at each *t* under different scenarios ($1-\alpha =95\%$ in red; $90\%$ in green; $80\%$ in blue).

As our main interest lies in the detection of change points, we first investigate the performance of the proposed Bayes estimator $\hat{\pmb {\delta }}$. We present the proportion of 100 simulated datasets where a change point is detected at time point *t* ($t=1,\ldots ,T$) in Fig. 4a–c. Under all three scenarios, the estimated change points are near the true ones, suggesting that the proposed Bayesian method can accurately locate the change points of a pandemic wave. Compared with the first two scenarios, the estimated change points under Scenario 3 are more concentrated around the truth. We also present the coverage probability of $(1-\alpha )$ probability regions (i.e., HPD intervals) of change points for each *t* under different scenarios and different values of $\alpha$ in Fig. 4d–f. The average length of HPD intervals is the shortest under Scenario 3, while intervals of the second and third change points sometimes intersect under Scenarios 1 and 2.

To quantitatively measure the agreement between the true $\pmb {\delta }$ (or $\pmb {\eta }$) and the estimator $\hat{\pmb {\delta }}$ (or $\hat{\pmb {\eta }}$), we transfer the stage allocation of time points $\{1,\ldots ,T\}$ to a clustering problem and adopt the adjusted Rand index^19^ and the mutual information^20^ as evaluation metrics.

- Adjusted Rand index (ARI):
  $$\begin{aligned} \text {ARI}=\frac{(\text {TP}+\text {TN})-\{(\text {TP}+\text {FP})(\text {TP}+\text {FN})+(\text {TN}+\text {FP})(\text {TN}+\text {FN})\}}{1-\{(\text {TP}+\text {FP})(\text {TP}+\text {FN})+(\text {TN}+\text {FP})(\text {TN}+\text {FN})\}}, \end{aligned}$$
  where the proportions of true positives (TP), false positives (FP), false negatives (FN) and true negatives (TN) in stage allocation are given by
  $$\begin{aligned} {\left\{ \begin{array}{ll} \text {TP}=\frac{2}{T(T-1)}\sum _{1\le t< t'\le T}{\mathbb {I}}(\eta _t=\eta _{t'},{\hat{\eta }}_t={\hat{\eta }}_{t'}),\\ \text {FP}=\frac{2}{T(T-1)}\sum _{1\le t< t'\le T}{\mathbb {I}}(\eta _t\ne \eta _{t'},{\hat{\eta }}_t={\hat{\eta }}_{t'}),\\ \text {FN}=\frac{2}{T(T-1)}\sum _{1\le t< t'\le T}{\mathbb {I}}(\eta _t=\eta _{t'},{\hat{\eta }}_t\ne {\hat{\eta }}_{t'}),\\ \text {TN}=\frac{2}{T(T-1)}\sum _{1\le t< t'\le T}{\mathbb {I}}(\eta _t\ne \eta _{t'},{\hat{\eta }}_t\ne {\hat{\eta }}_{t'}).\\ \end{array}\right. } \end{aligned}$$
  The range of ARI is [0, 1], where a larger ARI suggests that $\hat{\pmb {\delta }}$ is more similar to ${\pmb {\delta }}$ and the maximum possible value is obtained when $\hat{\pmb {\delta }}=\pmb {\delta }$.
- Mutual information (MI):
  $$\begin{aligned} \text {MI}=\sum _{k=1}^K\sum _{k'=1}^{{\hat{K}}} \frac{n_{kk'}}{T}\log \left( \frac{n_{kk'}T}{n_{k\cdot }n_{\cdot k'}}\right) , \end{aligned}$$
  where
  $$\begin{aligned} {\left\{ \begin{array}{ll} {\hat{K}}=\sum _{t=1}^{T}{\hat{\delta }}_t,\\ n_{kk'}=\sum _{t=1}^T{\mathbb {I}}(\eta _t=k,{\hat{\eta }}_t=k'),\\ n_{k\cdot }=\sum _{t=1}^T{\mathbb {I}}(\eta _t=k),\\ n_{\cdot k'}=\sum _{t=1}^T{\mathbb {I}}({\hat{\eta }}_t=k'). \end{array}\right. } \end{aligned}$$
  The range of MI is $[0,\sum _{k=1}^Kn_{k\cdot }/T\times \log (T/n_{k\cdot })]$, where a larger MI suggests that $\hat{\pmb {\delta }}$ is more similar to ${\pmb {\delta }}$ and the maximum possible value is obtained when $\hat{\pmb {\delta }}=\pmb {\delta }$. Under our three scenarios, the maximum possible value of MI is
  $$\begin{aligned} \sum _{k=1}^K\frac{n_{k\cdot }}{T}\times \log \left( \frac{T}{n_{k\cdot }}\right) =\sum _{k=1}^4 \frac{25}{100}\times \log \left( \frac{100}{25}\right) =\log 4=1.386, \end{aligned}$$
  because $$n_{k\cdot }=25$$ for $$k=1,\ldots ,4.$$

The performance of the Bayes estimator $\hat{\pmb {\delta }}$ under different scenarios is summarized in the first row of Table 1. It is clear that our estimator can accurately estimate the underlying $\pmb {\delta }$, especially under Scenario 3 with large changes of the transmission rate and removal rate between adjacent stages.

We also vary the hyper-parameter *p* to investigate how the performance of $\hat{\pmb {\delta }}$ is sensitive to the choice of prior probability. Both the ARI and MI of $\hat{\pmb {\delta }}$ are larger under $p=0.05$ than those under $p=0.01$ but with no significant gaps. This is because $p=0.05$ is closer to the true proportion of change points $K/T=0.04$ than 0.01 and thus true change points can be selected with more certainty, even though the signal strengths of true change points are still strong enough to be detected using $p=0.01$. When $p=0.2$, the ARI of $\hat{\pmb {\delta }}$ becomes smaller due to more false positives in change point detection. In general, the performance of $\hat{\pmb {\delta }}$ is stable as long as the prior probability *p* is not far larger than the true proportion of change points. A guideline to choose *p* is first checking the naive estimators of the transmission rate and removal rate (as discussed in the next section) and then taking the ratio of the number of change points and the study length *T*.

**Table 1:** Average values of accuracy measures of the Bayes estimator $\hat{\pmb {\delta }}$ with standard deviations in parentheses.

	ARI $\in [0,1]$			MI $\in [0,1.386]$		
	Scenario 1	Scenario 2	Scenario 3	Scenario 1	Scenario 2	Scenario 3
$p=0.01$	0.855 (0.092)	0.862 (0.094)	0.960 (0.035)	1.198 (0.107)	1.206 (0.107)	1.327 (0.049)
$p=0.05$	0.954 (0.039)	0.953 (0.037)	0.977 (0.023)	1.319 (0.054)	1.316 (0.052)	1.351 (0.035)
$p=0.2$	0.625 (0.040)	0.618 (0.042)	0.709 (0.050)	1.144 (0.057)	1.146 (0.051)	1.213 (0.058)

## Analysis of the Omicron wave in Singapore

**Figure 5:**
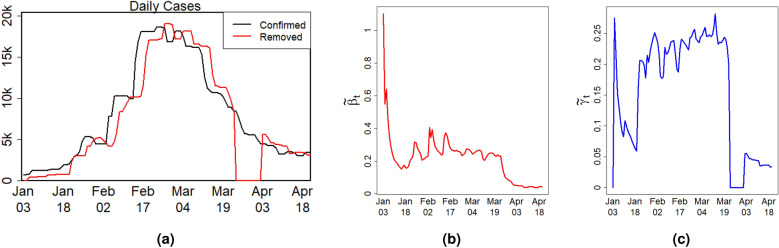
(**a**) The numbers of daily confirmed and removed COVID-19 cases, (**b**) the naive estimator of the time-varying transmission rate, and (**c**) the naive estimator of the time-varying removal rate during the Omicron wave in Singapore.

At the beginning of 2022, Singapore witnessed the outbreak of the Omicron wave of COVID-19 pandemic. To investigate change points of the Omicron wave, we collect the daily reported number of confirmed cases and removed cases in Singapore from January 3 to April 21, 2022, during which 893854 confirmed cases and 453 deaths of COVID-19 were reported in $T=109$ days. Let $N=5930134$ be the population size. The daily reported number of confirmed and removed cases during the study period is presented in Fig. 5a.

To gain insight into the trend of $\beta _t$ and $\gamma _t$, we compute the naive estimator of model (2),

$$\begin{aligned} {\tilde{\beta }}_t=-\frac{1}{P_{t-1}}\log \left( \frac{\Delta I_{t}}{S_{t-1}}\right) ,\quad {\tilde{\gamma }}_t=\frac{\Delta R_{t}}{I_{t-1}}, \quad {t=1,\ldots ,T}. \end{aligned}$$

As shown in Fig. 5b,c, the transmission rate was very high in the first several days and then became stable between January 18 and March 19, 2022. After March 19, the transmission rate decreased to almost zero. The removal rate was low before January 18, 2022. Between January 18 and March 23, the removal rate increased stably, which then had a sharp drop followed with a sudden increase because recovery cases were not reported from March 24 to April 1, 2022.

**Figure 6:**
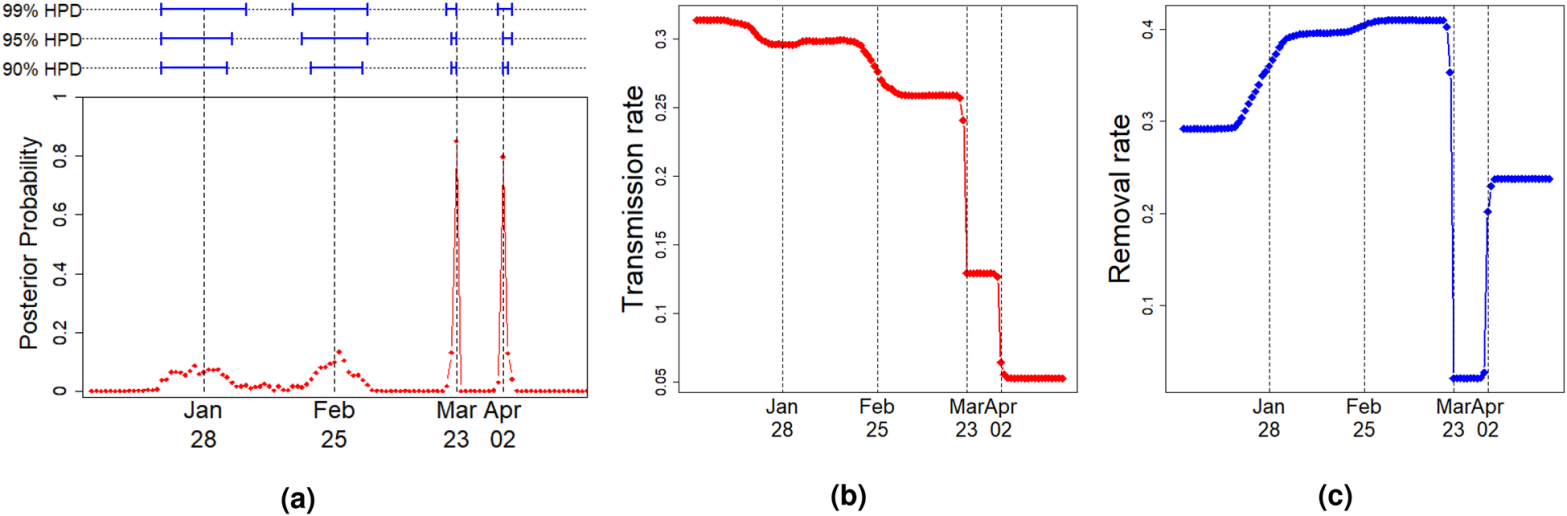
The Bayes estimator of (**a**) ${\pmb {\delta }}$ and 95% probability regions (HPD intervals) for change points; (**b**) the expected transmission rate; and (**c**) the expected removal rate.

Although the naive estimator suggests possible existence of change points in the study period, it cannot provide the estimates of change points with quantitative uncertainty measurement and thus we apply the proposed MCMC algorithm with hyperparameter $p=0.01$ to draw posterior samples of parameters for Bayesian inference. We discard the first 5000 iterations as burn-in and draw one posterior sample for every 10 iterations in the next 10000 iterations, leading to $G=1000$ posterior samples $(\pmb {\delta }^{(g)},{{\textbf {b}}}^{(g)},{{\textbf {r}}}^{(g)},\pmb {\beta }^{(g)},\pmb {\gamma }^{(g)})$ in total. The Bayes estimator $\hat{\pmb {\delta }}$ is presented in Fig. 6a, with estimated posterior probabilities $\sum _{g=1}^G \delta _t^{(g)}/G$ ($t=1,\ldots ,T$) and the corresponding probability regions of change points. Four change points are detected in the Omicron wave in Singapore. The probability regions of the third (March 23, 2022) and fourth (April 2, 2022) change points are shorter, while uncertainties of the first two change points (January 28 and February 25, 2022) are greater. With the smoothed estimators of the expected transmission rate and expected removal rate on day *t* ($t=1,\ldots ,T$) shown in Fig. 6b,c, we can interpret different stages as follows.

1. **January 3 to January 27**: The transmission rate was high while the removal rate was low, indicating the outbreak of the Omicron wave and medical resources in Singapore were not ready to cope with the Omicron variant yet.
2. **January 28 to February 24**: The transmission rate decreased slightly, indicating that people in Singapore had realized the outbreak of the Omicron wave and begun self-protection activities. In addition, the removal rate increased to a high level as the Ministry of Health in Singapore begun deploying medical resources to COVID-19 patients.
3. **February 25 to March 22**: The Omicron wave reached the peak and then passed it due to the sharp decrease in the transmission rate.
4. **March 23 to April 1**: An abnormal period was detected when recovery cases were not reported for several days, leading to a sharp drop in the estimated removal rate.
5. **April 2 to April 21**: The Omicron wave became stabilized and under control.

## Conclusion

The stochastic SIR model with change points is developed to quantify the evolvement of the Omicron wave of COVID-19 pandemic. The proposed model assumes that the numbers of daily confirmed cases and removed cases follow binomial distributions and the probability of a susceptible individual getting infected is determined by the proportion of currently infectious individuals and the time-varying transmission rate. We develop an MCMC algorithm to draw posterior samples and provide both the Bayes estimators and probability regions of change points, which are corroborated by experiments on simulated data. By analyzing the Omicron wave in Singapore, four change points are detected, corresponding to the increase of medical resources, decrease of the transmission rate, the beginning and the end of an abnormal period with no removal cases reported. The proposed model is applicable to a pandemic wave of any infectious disease under the impact of social changes. It is also possible to make inference on the spreading of COVID-19 and compare the effect of disease-control policies in different countries, as long as both daily numbers of confirmed cases and removal cases are available. In addition, our model can be easily extended to incorporate other interpersonal contact patterns into disease transmission. Although our model (2) is also applicable to sequential (real-time) change point detection in an ongoing epidemic, the goals for real-time detection are to minimize the detection delay as well as false detections of change points^21^. Timely detecting changes in the transmission rate and removal rate is critically important for policy making and identifying any possible new variants of the virus. To accommodate such objectives, construction of the posterior loss and Bayes estimators of change points warrant further investigation.

## Supplementary Information

Supplementary Information.

## Data Availability

All data generated and analyzed during this study are included in the supplementary file “Code.zip”.
